# Methyl 2-{[2-(4,4,5,5-tetra­methyl-1,3-dioxyl-4,5-dihydro­imidazol-2-yl)phen­yl]­oxy}acetate

**DOI:** 10.1107/S1600536811054018

**Published:** 2011-12-23

**Authors:** Hai-Bo Wang, Lin-Lin Jing, Xiao-Li Sun

**Affiliations:** a Department of Chemistry, School of Pharmacy, Fourth Military Medical University, Changle West Road 17, 710032, Xi-An, People’s Republic of China; b Department of Pharmacy, Lanzhou General Hospital of PLA, Key laboratory of the Prevention and Cure for the Plateau Environment Damage, PLA, 730050 Lanzhou Gansu, People’s Republic of China

## Abstract

In the title compound, C_16_H_21_N_2_O_5_, the benzene ring is nearly perpendicular to the imidazole ring, making a torsion angle of 88.6 (8)°·The crystal structure is stabilized by non-classical C—H⋯O and C—H⋯π inter­actions, which build up a three-dimensional network.

## Related literature

For the chemical and physical properties of nitronyl nitroxides, see: Osiecki & Ullman (1968 ▶). For their biological activity, see: Soule *et al.* (2007 ▶). For related structures, see: Wang *et al.* (2009 ▶); Jing *et al.* (2011 ▶). 
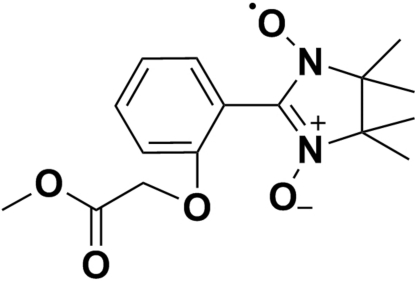


## Experimental

### 

#### Crystal data

C_16_H_21_N_2_O_5_
*M* *_r_* = 321.35
Monoclinic, 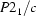

*a* = 11.421 (6) Å
*b* = 7.381 (4) Å
*c* = 19.700 (11) Å
β = 91.832 (6)°
*V* = 1659.8 (16) Å^3^
*Z* = 4
Mo *K*α radiation
μ = 0.10 mm^−1^
*T* = 296 K
0.23 × 0.21 × 0.14 mm

#### Data collection

Bruker APEXII CCD diffractometer
Absorption correction: multi-scan (*SADABS*; Bruker, 2007 ▶) *T*_min_ = 0.978, *T*_max_ = 0.987
11481 measured reflections
3082 independent reflections
1717 reflections with *I* > 2σ(*I*)
*R*_int_ = 0.049

#### Refinement

*R*[*F* ^2^ > 2σ(*F* ^2^)] = 0.060
*wR*(*F* ^2^) = 0.219
*S* = 0.95
3082 reflections
213 parameters
30 restraints
H-atom parameters constrained
Δρ_max_ = 0.42 e Å^−3^
Δρ_min_ = −0.42 e Å^−3^

### 

Data collection: *APEX2* (Bruker, 2007 ▶); cell refinement: *SAINT* (Bruker, 2007 ▶); data reduction: *SAINT*; program(s) used to solve structure: *SHELXS97* (Sheldrick, 2008 ▶); program(s) used to refine structure: *SHELXL97* (Sheldrick, 2008 ▶); molecular graphics: *ORTEP-3 for Windows* (Farrugia, 1997 ▶); software used to prepare material for publication: *SHELXTL* (Sheldrick, 2008 ▶) and *PLATON* (Spek, 2009 ▶).

## Supplementary Material

Crystal structure: contains datablock(s) I, global. DOI: 10.1107/S1600536811054018/hg5154sup1.cif

Structure factors: contains datablock(s) I. DOI: 10.1107/S1600536811054018/hg5154Isup2.hkl

Additional supplementary materials:  crystallographic information; 3D view; checkCIF report

## Figures and Tables

**Table 1:** Hydrogen-bond geometry (Å, °) *Cg*2 is the centroid of the phenyl ring.

*D*—H⋯*A*	*D*—H	H⋯*A*	*D*⋯*A*	*D*—H⋯*A*
C3—H3*A*⋯O4^i^	0.97	2.51	3.327 (5)	141
C3—H3*B*⋯O4^ii^	0.97	2.44	3.195 (5)	134
C8—H8⋯*Cg*2^iii^	0.93	2.99	3.896 (5)	164

Symmetry codes: (i) 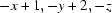; (ii) 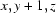; (iii) 
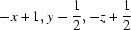
.

**Fig. 1.**
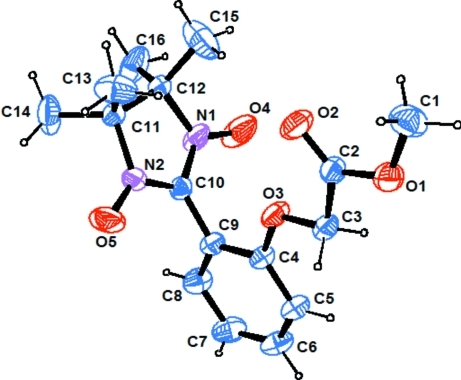
Molecular structure of the title compound with atom numbering scheme. Displacement ellipsoids are drawn at the 30% probability level. H atoms are presented as a small spheres of arbitrary radius.

## supplementary crystallographic information

### Comment

Nitronyl nitroxides, stable organic radicals, synthesized more than 30 years ago (Osiecki *et al.*, 1968), have received considerable attention recently because of their capability of magnetism, anticancer, antiradiation and antioxidation in biological chemistry and magnetic material fields (Soule *et al.* 2007).

The dihedral angle for imidazole and the phenyl rings is 88.6 (8)°. The dihedral angle is bigger than other nitronyl nitroxide reported on literature (Jing *et al.* 2011). In the title compound, the nitronyl nitroxide ring is almost in one plane, but the nitronyl nitroxide unit often displays a twisted or half-chair conformation for other related compounds (Wang *et al*.2009; Jing *et al.* 2011).

The crystal structure is stabilized by non-classical C—H···O and C—H···π (Table 1, *Cg*2 is the centroid of the phenyl ring) hydrogen bonds.

### Experimental

A mixture of 250 mg (1.0 mmol) of 2-(2'-Hydroxyl)phenyl-4,4,5,5-tetramethylimidazoline-3-oxide-1-oxyl, 0.30 ml of methyl bromoacetate and 100 mg of sodium methylate in 5 ml of anhydrous tetrahydrofuran was stirred at 60°C for 5 h and TLC (CHCl_3_/CH_3_OH, 20:1)indicated the complete disapperance of the raw material. Then the solvent was removed to give a dark blue residue which was purified by a flash column chromatography (eluent, chloroform and methanol, the ratio ofvolume is 20 to 1) to yield the title compound as a dark red powder. Single crystals of compound were obtained from the mixed solution of *n*-hexaneand dichloromethane (the ratio of volume is 1 to 1).

### Refinement

H atoms were positioned geometrically and were allowed to ride on the C atoms to which they are bonded, with C—H = 0.93–0.97 Å and *U*_iso_(H) = 1.2Ueq(C_aromatic_) or *U*_iso_(H) = 1.5Ueq(C_methyl_).

### Crystal data

**Table:**

C_16_H_21_N_2_O_5_	*F*(000) = 684
*M**_r_* = 321.35	*D*_x_ = 1.286 Mg m^−^^3^
Monoclinic, *P*2_1_/*c*	Mo *K*α radiation, λ = 0.71073 Å
Hall symbol: -P 2ybc	Cell parameters from 1441 reflections
*a* = 11.421 (6) Å	θ = 2.7–20.5°
*b* = 7.381 (4) Å	µ = 0.10 mm^−^^1^
*c* = 19.700 (11) Å	*T* = 296 K
β = 91.832 (6)°	Block, red
*V* = 1659.8 (16) Å^3^	0.23 × 0.21 × 0.14 mm
*Z* = 4	

### Data collection

**Table:**

Bruker APEXII CCD diffractometer	3082 independent reflections
Radiation source: fine-focus sealed tube	1717 reflections with *I* > 2σ(*I*)
graphite	*R*_int_ = 0.049
φ and ω scans	θ_max_ = 25.5°, θ_min_ = 2.1°
Absorption correction: multi-scan (*SADABS*; Bruker, 2007)	*h* = −13→13
*T*_min_ = 0.978, *T*_max_ = 0.987	*k* = −8→8
11481 measured reflections	*l* = −23→21

### Refinement

**Table:**

Refinement on *F*^2^	Primary atom site location: structure-invariant direct methods
Least-squares matrix: full	Secondary atom site location: difference Fourier map
*R*[*F*^2^ > 2σ(*F*^2^)] = 0.060	Hydrogen site location: inferred from neighbouring sites
*wR*(*F*^2^) = 0.219	H-atom parameters constrained
*S* = 0.95	*w* = 1/[σ^2^(*F*_o_^2^) + (0.140*P*)^2^] where *P* = (*F*_o_^2^ + 2*F*_c_^2^)/3
3082 reflections	(Δ/σ)_max_ < 0.001
213 parameters	Δρ_max_ = 0.42 e Å^−^^3^
30 restraints	Δρ_min_ = −0.42 e Å^−^^3^

### Special details

**Table:**

Geometry. All e.s.d.'s (except the e.s.d. in the dihedral angle between two l.s. planes) are estimated using the full covariance matrix. The cell e.s.d.'s are taken into account individually in the estimation of e.s.d.'s in distances, angles and torsion angles; correlations between e.s.d.'s in cell parameters are only used when they are defined by crystal symmetry. An approximate (isotropic) treatment of cell e.s.d.'s is used for estimating e.s.d.'s involving l.s. planes.
Refinement. Refinement of *F*^2^ against ALL reflections. The weighted *R*-factor *wR* and goodness of fit *S* are based on *F*^2^, conventional *R*-factors *R* are based on *F*, with *F* set to zero for negative *F*^2^. The threshold expression of *F*^2^ > σ(*F*^2^) is used only for calculating *R*-factors(gt) *etc*. and is not relevant to the choice of reflections for refinement. *R*-factors based on *F*^2^ are statistically about twice as large as those based on *F*, and *R*- factors based on ALL data will be even larger.

### Fractional atomic coordinates and isotropic or equivalent isotropic displacement parameters (Å^2^)

**Table:**

	*x*	*y*	*z*	*U*_iso_*/*U*_eq_	
C1	0.2619 (4)	1.4589 (6)	−0.1296 (2)	0.0838 (13)	
H1A	0.2219	1.3511	−0.1447	0.126*	
H1B	0.2999	1.5138	−0.1673	0.126*	
H1C	0.2065	1.5424	−0.1117	0.126*	
C2	0.3169 (3)	1.2963 (5)	−0.03053 (17)	0.0527 (9)	
C3	0.4199 (3)	1.2593 (4)	0.01652 (17)	0.0524 (9)	
H3A	0.4869	1.2222	−0.0092	0.063*	
H3B	0.4409	1.3678	0.0419	0.063*	
C4	0.4695 (3)	1.0655 (4)	0.10896 (18)	0.0509 (9)	
C5	0.5846 (3)	1.1206 (5)	0.1134 (2)	0.0616 (10)	
H5	0.6130	1.2036	0.0825	0.074*	
C6	0.6575 (3)	1.0515 (5)	0.1642 (2)	0.0705 (11)	
H6	0.7353	1.0887	0.1670	0.085*	
C7	0.6184 (3)	0.9304 (5)	0.2103 (2)	0.0749 (12)	
H7	0.6684	0.8865	0.2446	0.090*	
C8	0.5027 (3)	0.8732 (5)	0.2053 (2)	0.0667 (11)	
H8	0.4753	0.7893	0.2362	0.080*	
C9	0.4280 (3)	0.9395 (4)	0.15493 (18)	0.0505 (9)	
C10	0.3067 (3)	0.8761 (4)	0.14568 (16)	0.0461 (8)	
C11	0.1010 (3)	0.8621 (4)	0.14948 (16)	0.0476 (8)	
C12	0.1454 (3)	0.7110 (4)	0.10154 (17)	0.0505 (9)	
C13	0.0269 (4)	1.0061 (6)	0.1154 (2)	0.0898 (14)	
H13A	0.0068	1.0963	0.1482	0.135*	
H13B	−0.0434	0.9523	0.0965	0.135*	
H13C	0.0699	1.0616	0.0798	0.135*	
C14	0.0445 (5)	0.7921 (6)	0.2134 (2)	0.1018 (17)	
H14A	0.0916	0.6966	0.2330	0.153*	
H14B	−0.0324	0.7465	0.2020	0.153*	
H14C	0.0385	0.8891	0.2456	0.153*	
C15	0.1063 (6)	0.7353 (9)	0.0277 (2)	0.124 (2)	
H15A	0.1267	0.8547	0.0129	0.186*	
H15B	0.0230	0.7195	0.0233	0.186*	
H15C	0.1445	0.6470	0.0003	0.186*	
C16	0.1231 (4)	0.5185 (5)	0.1239 (3)	0.0999 (17)	
H16A	0.1644	0.4363	0.0954	0.150*	
H16B	0.0407	0.4934	0.1203	0.150*	
H16C	0.1501	0.5035	0.1702	0.150*	
N1	0.2724 (2)	0.7420 (4)	0.10481 (18)	0.0709 (10)	
N2	0.2136 (2)	0.9510 (4)	0.17210 (14)	0.0554 (8)	
O1	0.3482 (2)	1.4130 (3)	−0.07761 (13)	0.0671 (8)	
O2	0.2212 (2)	1.2324 (4)	−0.02691 (14)	0.0769 (9)	
O3	0.38813 (19)	1.1206 (3)	0.06102 (13)	0.0623 (7)	
O4	0.3397 (3)	0.6491 (5)	0.0681 (2)	0.1186 (12)	
O5	0.2140 (2)	1.0877 (5)	0.21176 (16)	0.1025 (12)	

### Atomic displacement parameters (Å^2^)

**Table:**

	*U*^11^	*U*^22^	*U*^33^	*U*^12^	*U*^13^	*U*^23^
C1	0.102 (3)	0.079 (3)	0.069 (3)	−0.011 (2)	−0.021 (2)	0.010 (2)
C2	0.053 (2)	0.052 (2)	0.053 (2)	−0.0063 (16)	0.0076 (16)	−0.0013 (17)
C3	0.0467 (18)	0.049 (2)	0.061 (2)	−0.0090 (15)	0.0073 (16)	0.0017 (17)
C4	0.0395 (17)	0.053 (2)	0.060 (2)	−0.0063 (15)	0.0010 (16)	0.0017 (17)
C5	0.046 (2)	0.058 (2)	0.080 (3)	−0.0111 (16)	0.0016 (18)	0.002 (2)
C6	0.046 (2)	0.063 (3)	0.101 (3)	−0.0059 (18)	−0.011 (2)	−0.003 (2)
C7	0.062 (2)	0.062 (2)	0.100 (3)	0.0017 (19)	−0.022 (2)	0.007 (2)
C8	0.067 (2)	0.058 (2)	0.075 (3)	−0.0052 (18)	−0.008 (2)	0.013 (2)
C9	0.0442 (17)	0.049 (2)	0.058 (2)	−0.0058 (15)	0.0019 (16)	−0.0013 (17)
C10	0.0486 (18)	0.0436 (19)	0.0463 (19)	−0.0044 (15)	0.0057 (15)	0.0049 (16)
C11	0.0458 (17)	0.0477 (19)	0.0496 (19)	−0.0064 (14)	0.0043 (14)	0.0001 (16)
C12	0.0446 (17)	0.054 (2)	0.053 (2)	−0.0072 (15)	0.0036 (15)	−0.0041 (17)
C13	0.084 (3)	0.071 (3)	0.112 (3)	0.021 (2)	−0.037 (3)	−0.017 (3)
C14	0.135 (4)	0.087 (3)	0.087 (3)	−0.029 (3)	0.064 (3)	−0.011 (3)
C15	0.146 (5)	0.161 (5)	0.065 (3)	0.050 (4)	−0.009 (3)	−0.027 (3)
C16	0.094 (3)	0.054 (3)	0.155 (5)	−0.019 (2)	0.056 (3)	−0.017 (3)
N1	0.0510 (16)	0.0576 (18)	0.106 (2)	−0.0131 (14)	0.0310 (16)	−0.0363 (17)
N2	0.0490 (16)	0.0664 (19)	0.0506 (17)	−0.0029 (14)	0.0012 (13)	−0.0165 (15)
O1	0.0691 (16)	0.0705 (17)	0.0612 (16)	−0.0152 (13)	−0.0052 (13)	0.0127 (13)
O2	0.0480 (15)	0.094 (2)	0.089 (2)	−0.0201 (14)	−0.0036 (13)	0.0184 (16)
O3	0.0472 (13)	0.0689 (17)	0.0704 (16)	−0.0189 (11)	−0.0041 (11)	0.0219 (13)
O4	0.0707 (18)	0.099 (2)	0.189 (3)	−0.0214 (15)	0.0467 (19)	−0.074 (2)
O5	0.076 (2)	0.124 (3)	0.107 (2)	−0.0052 (17)	−0.0016 (17)	−0.075 (2)

### Geometric parameters (Å, °)

**Table:**

C1—O1	1.439 (5)	C10—N1	1.328 (4)
C1—H1A	0.9600	C11—N2	1.498 (4)
C1—H1B	0.9600	C11—C13	1.503 (5)
C1—H1C	0.9600	C11—C14	1.524 (5)
C2—O2	1.194 (4)	C11—C12	1.557 (4)
C2—O1	1.324 (4)	C12—N1	1.468 (4)
C2—C3	1.500 (5)	C12—C16	1.512 (5)
C3—O3	1.403 (4)	C12—C15	1.518 (6)
C3—H3A	0.9700	C13—H13A	0.9600
C3—H3B	0.9700	C13—H13B	0.9600
C4—O3	1.365 (4)	C13—H13C	0.9600
C4—C5	1.376 (4)	C14—H14A	0.9600
C4—C9	1.392 (5)	C14—H14B	0.9600
C5—C6	1.378 (5)	C14—H14C	0.9600
C5—H5	0.9300	C15—H15A	0.9600
C6—C7	1.360 (5)	C15—H15B	0.9600
C6—H6	0.9300	C15—H15C	0.9600
C7—C8	1.388 (5)	C16—H16A	0.9600
C7—H7	0.9300	C16—H16B	0.9600
C8—C9	1.377 (5)	C16—H16C	0.9600
C8—H8	0.9300	N1—O4	1.272 (4)
C9—C10	1.468 (4)	N2—O5	1.276 (4)
C10—N2	1.320 (4)		
			
O1—C1—H1A	109.5	C13—C11—C12	115.2 (3)
O1—C1—H1B	109.5	C14—C11—C12	114.4 (3)
H1A—C1—H1B	109.5	N1—C12—C16	108.0 (3)
O1—C1—H1C	109.5	N1—C12—C15	106.5 (3)
H1A—C1—H1C	109.5	C16—C12—C15	110.1 (4)
H1B—C1—H1C	109.5	N1—C12—C11	101.7 (2)
O2—C2—O1	124.6 (3)	C16—C12—C11	115.8 (3)
O2—C2—C3	126.3 (3)	C15—C12—C11	113.9 (3)
O1—C2—C3	109.1 (3)	C11—C13—H13A	109.5
O3—C3—C2	107.8 (3)	C11—C13—H13B	109.5
O3—C3—H3A	110.2	H13A—C13—H13B	109.5
C2—C3—H3A	110.2	C11—C13—H13C	109.5
O3—C3—H3B	110.2	H13A—C13—H13C	109.5
C2—C3—H3B	110.2	H13B—C13—H13C	109.5
H3A—C3—H3B	108.5	C11—C14—H14A	109.5
O3—C4—C5	125.7 (3)	C11—C14—H14B	109.5
O3—C4—C9	114.3 (3)	H14A—C14—H14B	109.5
C5—C4—C9	120.0 (3)	C11—C14—H14C	109.5
C4—C5—C6	119.4 (4)	H14A—C14—H14C	109.5
C4—C5—H5	120.3	H14B—C14—H14C	109.5
C6—C5—H5	120.3	C12—C15—H15A	109.5
C7—C6—C5	121.6 (3)	C12—C15—H15B	109.5
C7—C6—H6	119.2	H15A—C15—H15B	109.5
C5—C6—H6	119.2	C12—C15—H15C	109.5
C6—C7—C8	119.0 (4)	H15A—C15—H15C	109.5
C6—C7—H7	120.5	H15B—C15—H15C	109.5
C8—C7—H7	120.5	C12—C16—H16A	109.5
C9—C8—C7	120.6 (4)	C12—C16—H16B	109.5
C9—C8—H8	119.7	H16A—C16—H16B	109.5
C7—C8—H8	119.7	C12—C16—H16C	109.5
C8—C9—C4	119.4 (3)	H16A—C16—H16C	109.5
C8—C9—C10	122.4 (3)	H16B—C16—H16C	109.5
C4—C9—C10	118.1 (3)	O4—N1—C10	125.1 (3)
N2—C10—N1	109.0 (3)	O4—N1—C12	120.3 (3)
N2—C10—C9	125.9 (3)	C10—N1—C12	114.5 (3)
N1—C10—C9	124.9 (3)	O5—N2—C10	125.8 (3)
N2—C11—C13	106.8 (3)	O5—N2—C11	121.0 (3)
N2—C11—C14	106.5 (3)	C10—N2—C11	113.2 (3)
C13—C11—C14	111.2 (3)	C2—O1—C1	117.2 (3)
N2—C11—C12	101.6 (2)	C4—O3—C3	117.7 (2)
			
O2—C2—C3—O3	6.6 (5)	N2—C10—N1—O4	175.5 (4)
O1—C2—C3—O3	−173.5 (3)	C9—C10—N1—O4	0.3 (6)
O3—C4—C5—C6	−179.1 (3)	N2—C10—N1—C12	−1.5 (4)
C9—C4—C5—C6	−0.8 (5)	C9—C10—N1—C12	−176.7 (3)
C4—C5—C6—C7	−0.2 (6)	C16—C12—N1—O4	61.9 (5)
C5—C6—C7—C8	1.0 (6)	C15—C12—N1—O4	−56.3 (5)
C6—C7—C8—C9	−0.7 (6)	C11—C12—N1—O4	−175.8 (4)
C7—C8—C9—C4	−0.3 (6)	C16—C12—N1—C10	−121.0 (4)
C7—C8—C9—C10	176.9 (3)	C15—C12—N1—C10	120.8 (4)
O3—C4—C9—C8	179.5 (3)	C11—C12—N1—C10	1.4 (4)
C5—C4—C9—C8	1.1 (5)	N1—C10—N2—O5	−177.4 (3)
O3—C4—C9—C10	2.2 (5)	C9—C10—N2—O5	−2.3 (6)
C5—C4—C9—C10	−176.2 (3)	N1—C10—N2—C11	1.0 (4)
C8—C9—C10—N2	93.2 (5)	C9—C10—N2—C11	176.1 (3)
C4—C9—C10—N2	−89.6 (4)	C13—C11—N2—O5	57.3 (4)
C8—C9—C10—N1	−92.4 (5)	C14—C11—N2—O5	−61.6 (4)
C4—C9—C10—N1	84.8 (4)	C12—C11—N2—O5	178.3 (3)
N2—C11—C12—N1	−0.7 (3)	C13—C11—N2—C10	−121.2 (3)
C13—C11—C12—N1	114.3 (3)	C14—C11—N2—C10	119.9 (3)
C14—C11—C12—N1	−115.0 (4)	C12—C11—N2—C10	−0.1 (3)
N2—C11—C12—C16	116.1 (3)	O2—C2—O1—C1	−3.0 (5)
C13—C11—C12—C16	−128.9 (4)	C3—C2—O1—C1	177.1 (3)
C14—C11—C12—C16	1.7 (5)	C5—C4—O3—C3	−7.2 (5)
N2—C11—C12—C15	−114.8 (4)	C9—C4—O3—C3	174.5 (3)
C13—C11—C12—C15	0.2 (5)	C2—C3—O3—C4	−179.6 (3)
C14—C11—C12—C15	130.9 (4)		

### Hydrogen-bond geometry (Å, °)

**Table:**

*Cg*2 is the centroid of the phenyl ring.

**Table:**

*D*—H···*A*	*D*—H	H···*A*	*D*···*A*	*D*—H···*A*
C3—H3A···O4^i^	0.97	2.51	3.327 (5)	141
C3—H3B···O4^ii^	0.97	2.44	3.195 (5)	134
C8—H8···Cg2^iii^	0.93	2.99	3.896 (5)	164

Symmetry codes: (i) −*x*+1, −*y*+2, −*z*; (ii) *x*, *y*+1, *z*; (iii) −*x*+1, *y*−1/2, −*z*+1/2.
